# Processing Prescriptively Incorrect Comparative Particles: Evidence From Sentence-Matching and Eye-Tracking

**DOI:** 10.3389/fpsyg.2020.00186

**Published:** 2020-02-14

**Authors:** Ferdy Hubers, Theresa Redl, Hugo de Vos, Lukas Reinarz, Helen de Hoop

**Affiliations:** ^1^Centre for Language Studies, Radboud University Nijmegen, Nijmegen, Netherlands; ^2^Max Planck Institute for Psycholinguistics, Nijmegen, Netherlands; ^3^Institute of Public Administration, Leiden University, Leiden, Netherlands; ^4^Department of Physics, University of Bonn, Bonn, Germany

**Keywords:** grammatical norm violations, comparative particles, sentence-matching, eye-tracking, grammaticality

## Abstract

Speakers of a language sometimes use particular constructions which violate prescriptive grammar rules. Despite their prescriptive ungrammaticality, they can occur rather frequently. One such example is the comparative construction in Dutch and similarly in German, where the *equative* particle is used in comparative constructions instead of the prescriptively correct *comparative* particle (Dutch *beter als Jan* and German *besser wie Jan* “lit. better as John”). In a series of three experiments using sentence-matching and eye-tracking methodology, we investigated whether this grammatical norm violation is processed as grammatical, as ungrammatical, or whether it falls in between these two. We hypothesized that the latter would be the case. We analyzed our data using linear mixed effects models in order to capture possible individual differences. The results of the sentence-matching experiments, which were conducted in both Dutch and German, showed that the grammatical norm violation patterns with ungrammatical sentences in both languages. Our hypothesis was therefore not borne out. However, using the more sensitive eye-tracking method on Dutch speakers only, we found that the ungrammatical alternative leads to higher reading times than the grammatical norm violation. We also found significant individual variation regarding this very effect. We furthermore replicated the processing difference between the grammatical norm violation and the prescriptively correct variant. In summary, we conclude that while the results of the more sensitive eye-tracking experiment suggest that the grammatical norm violation is not processed completely on a par with ungrammatical sentences, the results of all three experiments clearly show that the grammatical norm violation cannot be considered grammatical, either.

## Introduction

Decades of experimental research contrasting grammatical with ungrammatical sentences have taught us much about language processing (e.g., Hagoort et al., 1993; Friederici et al., 2006). But what exactly constitutes an ungrammatical sentence? To a linguist, a grammatical sentence is one that adheres to the natural rules and constraints of a native speaker’s grammar, produced and understood by those exposed to the same input; ungrammatical sentences are constructions that are in principle not generated by a native speaker’s competence, although grammaticality judgments may vary (Schütze, 1996). To many language users, in contrast, an ungrammatical sentence is one that is prescriptively “incorrect” and is not, or rather in their view should not, be part of the standard language. These definitions clash when considering constructions that are frequently produced and encountered by native speakers of the language, yet nevertheless firmly disapproved of by speakers who adhere to prescriptive grammar rules. Consider the following sentence in (1), a well-known example of such a construction in Dutch (Hubers and de Hoop, 2013).


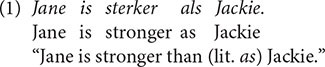


In (1) the use of *als* “as” instead of *dan* “than” is deemed incorrect by speakers of Dutch, even though prescriptive language guides often acknowledge the existence of this variant in a comparative construction (van der Meulen, 2018). Comparatives in Dutch (and German) belong to the category of “linguistic constructions where at least some degree of unwanted variation exists or is thought to exist” (van der Meulen, 2018: 79). Prescriptively disapproved variants such as *als* “as” in (1) are called *grammatical norm violations* (Hubers et al., 2016) or *grammatical taboos* (Vogel, 2019).

It remains unclear how such grammatical norm violations are processed by the human brain. Do they pattern with grammatical sentences, do they pattern with ungrammatical sentences, or do they fall in between grammatical and ungrammatical constructions when it comes to processing? The present paper employs two experimental techniques to investigate the online processing of grammatical norm violations compared to the processing of both grammatical and ungrammatical sentences. Before we continue to discuss these experiments in Sections “Experiments 1 and 2: Sentence-Matching” and “Experiment 3: Eye-Tracking,” Section “Grammaticality vs. Acceptability” reviews literature on (the processing of) ungrammaticality vs. unacceptability, focusing on one particular grammatical norm violation in Dutch (and German), namely, the use of an equative particle in a comparative construction as illustrated in (1) above.

## Grammaticality Vs. Acceptability

In an acceptability judgment study, Vogel (2019) found that grammatical taboos are judged as marked, but not to the same degree as truly ungrammatical sentences. He carried out three different types of acceptability tasks, one asking for aesthetic judgments, one for normative judgments (i.e., whether the construction was considered prescriptively correct), and one for possibility (i.e., estimated occurrence frequency). The grammatical taboos were judged in between grammatical (unmarked) filler sentences and ungrammatical ones, and behaved approximately on a par with the linguistically marked filler sentences in all three types of judgment experiments. Both types of markedness received a similar mean value in the different acceptability tasks, although grammatical taboos were disapproved of more strongly than linguistically marked sentences under the aesthetic judgment test. The two most salient grammatical taboos even grouped together with the ungrammatical sentences under the aesthetic judgment test. Yet, what Vogel considered the strongest grammatical taboo in his study, that is, the use of the verb *tun* “do” as an auxiliary in German, overall received a significantly higher acceptability rate than the ungrammatical filler sentences. In fact, it even came out as grammatical under the possibility type of judgment test.

Vogel (2019) also raised the question whether his empirical method could distinguish between grammatical taboos and linguistically marked sentences, based on their source of markedness. The markedness of linguistically marked sentences has a grammar-internal cause, whereas the markedness of grammatical taboos is caused externally, namely, by a social norm and about ten years of education. Vogel (2019) indeed found a difference between the two types of markedness (internally or externally caused), namely, in the between-subject variance. Participants were more uniform in their judgments of linguistically marked sentences than in their judgments of grammatical taboos. Clearly, grammatical taboos are not always marked or unacceptable for everybody, as there are many speakers who actually use these constructions themselves, perhaps even unaware of their low sociolinguistic status in prescriptive grammar.

Focusing on language users who are definitely aware of this lower prestige, Hubers et al. (2016) set up an fMRI study in which they presented sentences containing grammatical norm violations as well as grammatical and ungrammatical sentences. Their participants were recruited on the basis of their knowledge of prescriptive grammar rules, but also because of their strong negative attitude toward grammatical norm violations. To test whether social cognition was involved in the processing of grammatical norm violations, Hubers et al. also compared grammatical norm violations to sentences describing violations of social norms in their experiment. The latter type of sentences did not contain a linguistic violation, hence were grammatical. The authors did not find any effects specific to the processing of grammatical norm violations, whereas they did so for social norm violations. Also, during the processing of grammatical norm violations, some brain regions were activated that were also involved in the processing of ungrammatical sentences. No evidence for overlapping brain regions was found for social norm violations in comparison with ungrammatical sentences. These results suggest that grammatical norm violations are purely linguistic violations and not social ones. Still, grammatical norm violations are not completely ungrammatical, since Hubers et al. (2016) also found that similar brain regions were involved in both the processing of grammatical sentences and grammatical norm violations. Their explanation was that both types of sentences can be interpreted and integrated with conceptual memory equally well.

The present paper further investigates the online processing of grammatical norm violations compared to the processing of both grammatical and ungrammatical sentences using two other experimental techniques than Hubers et al. (2016). The first method we apply is a sentence-matching task, first used by Forster (1979). In a sentence-matching task, participants are sequentially presented with two sentences, and they have to indicate whether the second sentence is identical to the first one or not. Identical grammatical sentences are confirmed faster than identical ungrammatical sentences (Forster, 1979; Freedman and Forster, 1985; Forster and Stevenson, 1987; Duffield et al., 2002, 2007). Duffield et al. (2007) use the task to investigate a French construction that is deemed ungrammatical, but that is processed in the same way as grammatical sentences. Duffield et al. conclude that the construction is underlyingly grammatical. Hubers et al. (2016), on the other hand, found an increased activation in Inferior Frontal Gyrus for grammatical norm violations, similar to the activation found for ungrammatical sentences as opposed to grammatical sentences (Hagoort, 2005; Friederici et al., 2006; Snijders et al., 2009), but they also found processing overlap between grammatical norm violations and truly grammatical sentences. On the basis of the elicited reaction times, the sentence-matching task provides a straightforward method to find out whether grammatical norm violations as in (1) above are processed as either grammatical or ungrammatical, or indeed somewhere in between. The latter result could be concluded from reaction times slower than those for ungrammatical sentences but faster than those for grammatical and prescriptively correct ones. This would indicate an intermediate grammaticality status, in accordance with the results of Hubers et al. (2016) and Vogel (2019). The second method we apply is eye-tracking. This paradigm can give more information on the time course of the processing of the grammatical norm violation. If the ungrammaticality judgment of a grammatical norm violation is the result of a more conscious process than in the case of truly ungrammatical sentences, then we may expect the processing difficulties that arise with grammatical norm violations to be more global and to occur only later in the reading process. Therefore, tracking the eye-movements of participants incrementally reading these grammatical norm violations in comparison with reading grammatical and ungrammatical sentences will provide a valuable addition to the overall reaction time data from the sentence-matching task.

In the current paper, the grammatical norm violation under study concerns the use of an equative particle in a comparative construction. We focus on this particular grammatical norm violation, because it is one of the few violations that are prominent in both Dutch and German. Moreover, we did not include other grammatical norm violations in our study, since these were not expected to lead to different results, as was also shown in a *post hoc* analysis by Hubers et al. (2016). No processing differences were found between the various grammatical norm violations included in their study.

Before we continue to discuss these two types of experiments in Sections “Experiments 1 and 2: Sentence-Matching” and “Experiment 3: Eye-Tracking”, the remainder of Section “Grammaticality vs. Acceptability” reviews the particular grammatical norm violation in Dutch (and German) that constitutes the focus of the present paper, the use of an equative particle in a comparative construction, as illustrated in (1) above.

### Grammaticality vs. Acceptability in Comparative Particles in Dutch and German

Recall example sentence in (1) above, repeated below for convenience, which reflects the Dutch transition from the use of the comparative particle *dan* “than” toward the equative particle *als* “as” in comparatives (Reinarz et al., 2016).


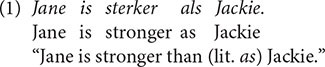


In present-day German a similar process takes place, in which the equative particle *wie* “as” is used in comparatives, instead of the comparative particle *als* “than” (Jäger, 2010). Whereas different theoretical linguistic analyses have been proposed to account for this replacement of a comparative particle by an equative particle in comparatives (cf. Postma, 2006; Reinarz et al., 2016; Jäger, 2019), the underlying idea of all of these theoretical analyses is that there must be an important grammar-internal factor in the grammars of Dutch and German inducing it. Postma (2006) argues that this factor is that the particle *dan* “than” has lost its original negative meaning. Reinarz et al. (2016) argue that the replacement results from a conflict between two competing functional principles, Economy and Iconicity. Jäger (2019) proposes a syntactic reanalysis of comparison constructions as embedded clauses, on the basis of a historically underlying correlative construction. All of these analyses thus assume the change to be internal to the language system, taking place irrespective of external (counter)forces. However, as Milroy and Milroy (1985: 348) state, “some innovations may not be accepted by a community and hence may not lead to change.” This adequately characterizes the current state of affairs concerning the use of an equative particle in a comparative in Dutch and German. Hubers and de Hoop (2013) find a strong correlation between level of education and the use of *als* “as” or *dan* “than” in a comparative. They argue that this correlation clearly reflects the strong influence of the prescriptive rule taught in schools (see also Hubers et al., 2019), repressing the use of an equative particle in a comparative construction in Dutch. The prescriptive rule against the use of an equative particle in a comparative construction is a well-known issue in German, too (Grebe, 1966; Jäger, 2010). To sum up, on the one hand, the use of an equative particle in a comparative can be considered a linguistic innovation, which is somehow caused language-internally. On the other hand, the use of a comparative particle in a comparative reflects a language-external prescriptive rule, as prescriptivists are notoriously intolerant of innovations in language. The result of these two counterforces, one language-internal and one language-external, is the extant variation up until now between two particles in comparatives in both Dutch and German.

Whatever motivates the use of an equative particle in a comparative construction in Dutch and German, the fact that it frequently occurs in the language makes this grammatical norm violation different from ordinary ungrammatical sentences, which hardly ever show up in everyday speech. Not only is the prescriptive rule explicitly taught in secondary education (Hubers et al., 2019), lay people also regularly express their concerns about the grammatical norm violation on social media (cf. Ermans, 2016 on German). The following anecdote from January 2014 serves to illustrate. In a radio interview, the former chair of the Dutch parliament, Anouchka van Miltenburg, said that each day when she got up, she decided to do her job better *als gisteren* “as yesterday.” This grammatical norm violation gave rise to so many negative reactions from the audience, especially on Twitter, that when the interview was broadcasted again the next day, one could hear van Miltenburg all of a sudden say that she would do her job better *dan gisteren* “than yesterday.” The recorded audio material of the interview had been edited, and van Miltenburg’s *als* “as” had been cut out and replaced by *dan* “than.”^1^

On the basis of Hubers et al. (2016), we expect to find processing differences between the grammatical norm violations (Dutch *beter als* and German *besser wie* “better as”) and the ungrammatical constructions (Dutch *beter wie* and German *besser wer*, “better who”), as well as between grammatical norm violations and their grammatical and prescriptively correct counterparts (Dutch *beter dan* and German *besser als* “better than”). More specifically, we expect that the processing cost due to the grammatical norm violation is smaller and less immediate than the processing cost caused by an ungrammatical construction. This is because the latter is caused by a language-internal type of ungrammaticality, whereas the processing cost linked to the grammatical norm violation is caused externally, by a prescriptive norm, and thus may emerge only after the subject’s conscious evaluation of the construction. We will also explore individual differences in the processing of the three constructions.

## Experiments 1 and 2: Sentence-Matching

We conducted two versions of the sentence-matching experiment in order to investigate the processing of grammatical norm violations. Experiment 1 was conducted in Dutch with native speakers of Dutch and Experiment 2 was conducted in German with native speakers of German.

### Materials and Methods

#### Participants

In total, 38 university students participated in Experiment 1 (seven males). They were all native speakers of Dutch, and most of them (35) were right-handed. Participants were recruited through SONA, the participant database of Radboud University. They all had normal or corrected-to-normal vision. The experiment took about 30 min and participants were rewarded with a 5 Euro gift card or course credit.

A total of 92 German university students participated in Experiment 2 (24 males). The data were collected at Radboud University in the Netherlands, University of Cologne, and Free University Berlin in Germany. The majority of the participants were right-handed (81) and all of them had normal or corrected-to-normal vision. Just like Experiment 1, Experiment 2 took about 30 min. Participation was compensated with a 5 Euro gift card.

#### Materials

The materials in Experiment 1 consisted of 18 experimental sentences and 76 filler sentences. The experimental sentences all included a comparative construction, and were taken from Hubers et al. (2016). Three versions of the same experimental sentence were created by changing the comparative particle. In the first version, the equative particle *als* “as” was used, resulting in a grammatical norm violation [condition GN, see (2a)]. The second version contained the grammatical and prescriptively correct particle *dan* “than” [condition GC, presented in (2b)], and the third version contained the question word *wie* “who,” leading to a truly ungrammatical sentence [condition UG, see (2c)]. The experimental sentences were all presented in the matching condition, requiring a yes response.


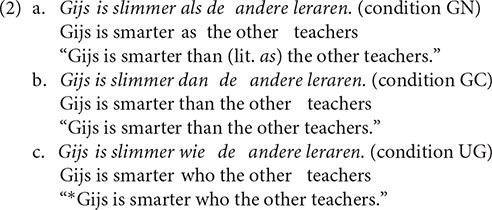


As reported in Hubers et al. (2016), the experimental sentences were all pretested to see whether they elicited the intended effect. A grammaticality judgment task revealed that more than 80% of the 136 participants judged the grammatical sentences as being grammatically correct, while less than 20% of the participants judged the ungrammatical sentences as being grammatically correct.

The fillers were all grammatical sentences. Based on Duffield et al. (2002), about 40% of the fillers were presented in the non-matching condition, leading to one-third of all materials requiring a no-response. To this end, we created slightly adapted versions of the sentences by changing only one word. An example of a filler sentence pair in the non-matching condition can be found in (3).


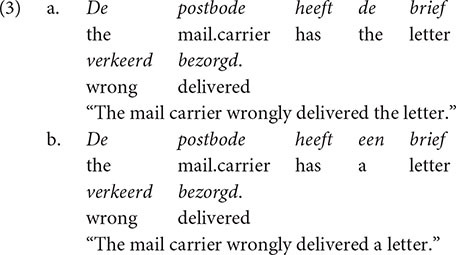


In order to control for sentence length, both the filler and experimental sentences consisted of 12 or 13 syllables.

In Experiment 2, the same sentences were used as in Experiment 1, but translated into German by a German-Dutch bilingual student. See (4a–c) for the German equivalents of the Dutch experimental sentences presented in (2). Dutch proper names were replaced by German ones.


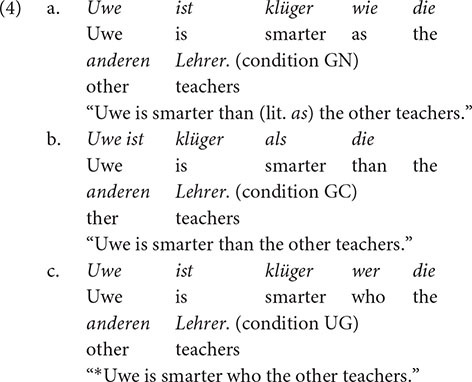


Fillers were also translated into German, see (5) for the translation of example (3).


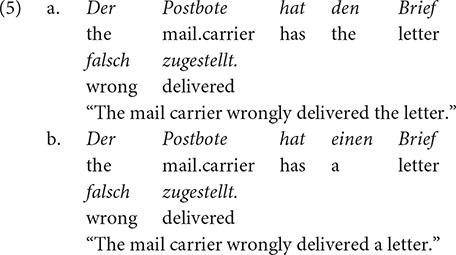


Sentence length of the filler and experimental sentences in Experiment 2 were comparable to those of Experiment 1.

#### Procedure

The procedure of Experiments 1 and 2 was identical. Participants were tested in a sound-attenuated booth. The experiment was conducted using E-prime (Schneider et al., 2002), and a buttonbox was used to record the participants’ responses.

A trial started with a fixation cross that was presented at the center of the screen for 250 ms. Subsequently, the first sentence was displayed at the top left of the screen for 3000 ms. Next, the second sentence was presented at the bottom of the screen. Upon the presentation of the second sentence, participants were instructed to indicate as quickly as possible whether the second sentence was identical to the first one by using the buttonbox. The button corresponding with “yes” was always located at the dominant hand. The second sentence disappeared after an answer was given by the participant or after 3000 ms.

The experiment started with a practice session consisting of six practice trials in order for the participant to get used to the task. After the practice session, they had the opportunity to ask questions if anything was unclear. Subsequently, participants completed two blocks of 47 trials separated by a short break. After the experiment, they filled in a background questionnaire. In addition, participants were presented with 10 sentences which they had to correct if necessary. The aim of this test was to assess participants’ familiarity with Dutch prescriptive grammar rules.

#### Analysis

We analyzed the data of Experiments 1 and 2 using linear mixed effects models in R with the lme4 package (Bates et al., 2015). The reaction time data were log transformed to correct for a right skew in the data. The three-level factor *condition* (*als/wie* “as,” *dan/als* “than,” *wie/wer* “who”) was coded using simple contrasts (UCLA and Statistical Consulting Group, 2011). With simple contrasts, the reference level is always coded as −1/3, and the level that is contrasted is coded as 2/3. They are similar to treatment contrasts, but have the advantage that the intercept corresponds to the mean of means instead of corresponding to the mean of the reference level. One contrast compared *als/wie* “as” to *dan/als* “than” (i.e., contrast 1, coded as −1/3, 2/3, −1/3), the other compared *als/wie* “as” to *wie/wer* “who” (i.e., contrast 2, coded as −1/3, −1/3, 2/3). We included random intercepts for items and participants, as well as random slopes for condition for both items and participants. Following Bates et al. (2015), overparameterization was checked by means of principal component analysis. In case of overparameterization, correlation parameters were dropped as a first step. If overparameterization persisted, individual variance components were removed. However, this later step was never necessary, and all models were fitted with the full random structure excluding correlation parameters. *P*-values were obtained using the package lmerTest (Kuznetsova et al., 2017). Finally, in order to gauge the individual variation between participants, we carried out two different model comparisons. We compared the full model to a model without random slopes for contrast 1 per participant, and we also compared the full model to a model without random slopes for contrast 2 per participant. This was done to see whether the two variance components significantly increased the model fit. In other words, we tested whether individual variation was significant.

### Results

#### Experiment 1

**FIGURE 1:**
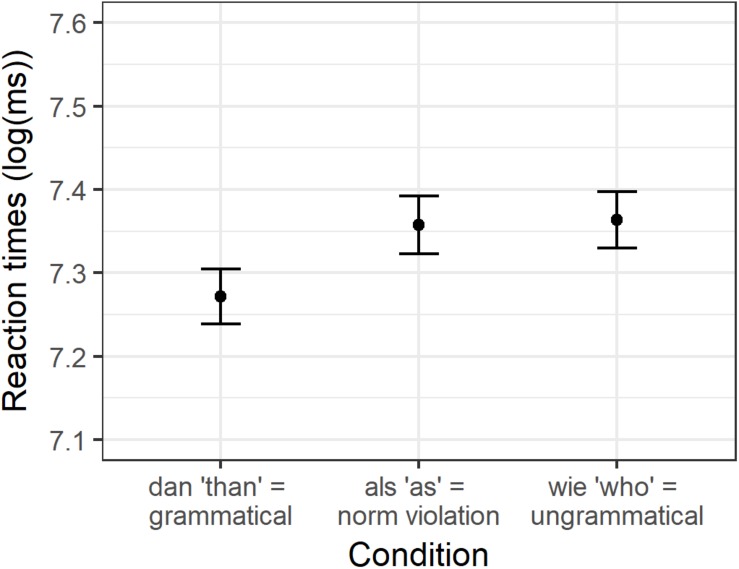
Condition means for logarithmically transformed reaction times in Experiment 1.

The analyses were conducted on the correct responses on the experimental sentences only. To this end, 5.5% of the data was removed. The averaged logarithmically transformed reaction times and standard deviations of the experimental sentences per condition are visualized in Figure 1. The mean reaction times and SDs on the response scale (in milliseconds) are presented in Supplementary Table S1.

The linear mixed effects regression analysis revealed a significant effect of *als* vs. *dan* (beta = −0.09, SE = 0.022, *t* = −3.87, *p* < 0.01). Participants took longer to decide that the sentences were identical if the sentences contained the grammatical norm violation (*als* “as”) as compared to its grammatically correct counterpart (*dan* “than”). Decisions to sentence pairs containing *als* “as” did not significantly differ from decisions to sentences containing *wie* “who” (beta = 0.01, SE = 0.024, *t* = 0.318, *p* = 0.75).

##### Gauging individual differences

As can be seen from Table 1, adding random slopes per participant for the contrast comparing *als* and *wie* did not significantly improve the full model. The same result was found for random slopes per participant for the contrast comparing *als* and *dan*. This suggests no significant individual variation among participants.

**TABLE 1:** Results of significance tests for random slope components per participant in Experiment 1.

	**Chi squared (*df* = 1)**	***p*-value**
*Contrast 1 (als vs. dan)*	0	1
*Contrast 2 (als vs. wie)*	0.132	0.717

#### Experiment 2

**FIGURE 2:**
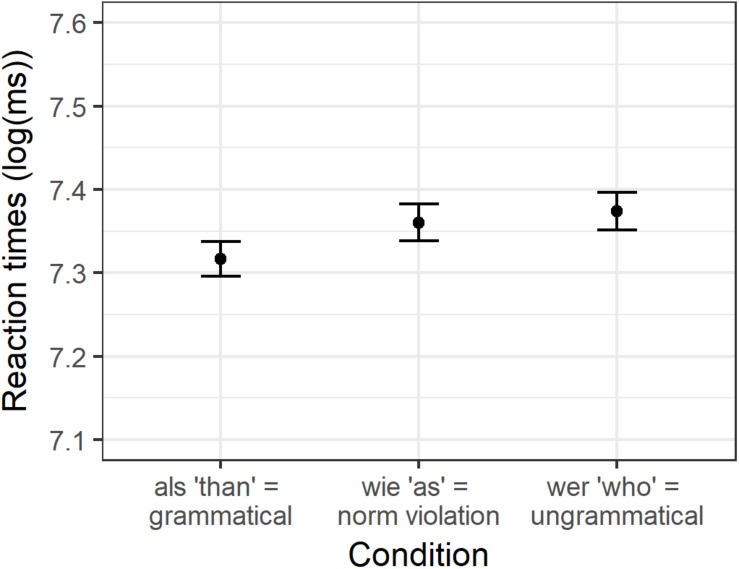
Condition means for logarithmically transformed reaction times in Experiment 2.

As in Experiment 1, only the experimental sentences were included in the analyses that were responded to correctly. Therefore, 5.7% of the data had to be removed. Figure 2 displays the average logarithmically transformed reaction times and the corresponding SDs for the experimental items per condition. The mean reaction times and SDs on the response scale are presented in Supplementary Table S2.

The analysis showed a significant effect between *wie* “as” and *als* “than” (beta = −0.04, SE = 0.019, *t* = −2.181, *p* < 0.05). More specifically, German participants were slower to decide that a sentence pair was identical if it contained a grammatical norm violation (the particle *wie* “as”) than if it was grammatical. No significant difference was observed between sentences containing a grammatical norm violation (*wie* “as”) and the ungrammatical *wer* “who” (beta = 0.02, SE = 0.015, *t* = 1.127, *p* = 0.27).

##### Gauging individual differences

Similar to Experiment 1, the model did not significantly improve after adding random slopes per participant for the contrast comparing *wie* “as” and *als* “than” and random slopes per participant for the contrast comparing *wie* “as” and *wer* “who” (see Table 2).

**TABLE 2:** Results of significance tests for random slope components per participant in Experiment 2.

	**Chi squared (*df* = 1)**	***p*-value**
*Contrast 1 (wie vs. als)*	0.016	0.9
*Contrast 2 (wie vs. wer)*	1.597	0.206

### Discussion

Unlike what we had predicted on the basis of Hubers et al. (2016), we did not find a difference between the processing of the grammatical norm violation *beter als/besser wie* “better as” and the ungrammatical condition *beter wie/besser wer* “better who,” whereas the processing of the grammatical norm violation differed significantly from the processing of its grammatical and prescriptively correct counterpart *beter dan/besser als* “better than.” Apparently, when participants have to determine whether two sentences are identical, the grammatical norm violation slows down this process to the same extent as the ungrammatical sentence. If it is true that a sentence-matching task provides us with a better measure of grammaticality than a grammatical judgment task, as claimed by Duffield et al. (2002, 2007), we must conclude that at least this particular grammatical norm violation is not underlyingly grammatical, but plainly ungrammatical.

However, in a sentence-matching task, the processing of a sentence is measured only after the full sentence has been read and can be judged. We assume that our participants, who were mostly university students, are well aware of the grammatical norm violation when they encounter it. Yet, it may be that this realization does not arise immediately. That is, if the grammatical norm violation is in fact underlyingly grammatical, processing will not be hampered immediately, but rather only when the linguistic awareness has arisen that the sentence is a grammatical norm violation. In order to find out whether processing a grammatical norm violation is comparable to processing a grammatical sentence in its initial stage of processing, we decided to conduct an eye-tracking experiment. We hypothesize that grammatical sentences will be processed with most ease, and that grammatical norm violations will possibly pattern with them. Ungrammatical sentences will lead to the largest processing cost.

## Experiment 3: Eye-Tracking

### Materials and Methods

#### Participants

The data were collected as part of another experiment, for which our stimuli functioned as fillers. Due to the design of this other experiment, participants were not equally distributed over our three lists, leading to, respectively, 45, 45, and 30 participants. We excluded 22 participants that grew up with the Limburgian dialect, or had a background in linguistics. We excluded Limburgian participants because in their dialect *wie* “who” as a particle in comparative constructions does occur. Participants with a background in linguistics were excluded because they might be aware of the phenomenon of grammatical norm violations. After excluding these participants, we were left with data of 36 participants for lists 1 and 2, and data of 26 participants for list 3. Subsequently, we randomly excluded 10 participants from lists 1 and 2 to match the number of participants in list 3. This led to a total of 78 participants (31 male) to be included in the analysis. These participants answered at least 75% of correction questions pertaining to filler items correctly. The participants ranged in age from 18 to 28 (M = 21.8) and were all native speakers of Dutch. Participants were recruited through SONA, the participant database of Radboud University. All participants had normal or corrected-to-normal vision. The experiment took approximately one hour, and reimbursement was a 10 Euro coupon or course credit, if preferred.

#### Materials

Each participant saw 18 stimuli in three conditions. These stimuli were based on the experimental sentences included in the Dutch version of the sentence-matching experiment (Experiment 1). Each item occurred in every condition, and three lists were created to be equally distributed across participants.

#### Procedure

The experiment was conducted at the Centre for Language Studies labs at Radboud University. We used an EyeLink 1000 + remote desktop eye-tracker with a chinrest to stabilize the participants’ head. Viewing was binocular and the participants’ dominant eye was sampled at 1000 Hz. If it was not possible to sample the dominant eye (e.g., due to glare which often occurs when participants wear glasses), the other eye was measured. This was the case for 14 participants. The stimuli were presented at a distance of 108 cm on a BenQ XL 2420T 24” LED using Experiment Builder by SR Research. The stimuli were presented in 19-point Calibri font on a light gray background. Upon arrival, participants were given an information document about the experiment and asked to sign a consent form. The experimenter then determined the participant’s dominant eye. They were then accompanied to the testing booth where they read the instructions. The experimenter then set up the eye-tracker and made sure participants were comfortable. Participants then performed a 13-point calibration and validation. They then saw four practice items, after which they got the opportunity to ask questions. After another calibration routine, the experiment started. Participants got the opportunity to take breaks after one- and two-thirds of the experiment. After the experiment, participants filled in a short questionnaire, probing participants for the purpose of the experiment. Finally, they were paid.

#### Analysis

The raw eye-tracking data were pre-processed using EyeLink Data Viewer by SR Research. Using this software, we examined the fixation pattern of each item for each participant. The fixations were reassigned in case a clear shift had occurred. Furthermore, we deleted the first fixation of a trial if it did not fall on the first line of the stimulus. Fixations that were smaller than 80 ms were merged with another fixation within 0.25 degrees (i.e., within 0.47 cm) in visual angle on the *x*-axis if this fixation exceeded 80ms. Subsequently, unmerged fixations that were larger than 1200 ms or smaller than 80 ms were deleted. We calculated three reading time measures for the regions of interest: first run dwell time (i.e., the sum of the duration of all fixations in a region when it is entered for the first time), regression path duration (i.e., first run dwell time with the addition of the duration of fixations back to previous regions out of the analyzed region), and dwell time (i.e., the sum of the duration of all fixations in a region, also known as total fixation duration). The two regions of interest were defined as follows, as indicated by the square brackets:


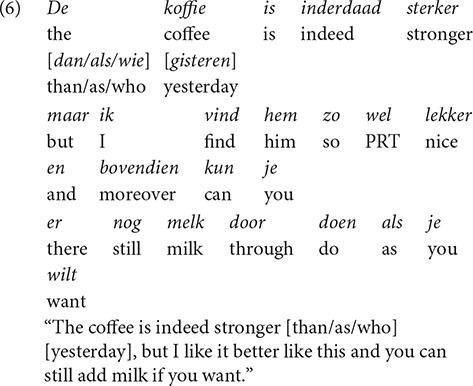


The comparative particle was always followed by the word *gisteren* “yesterday” in order to keep the spillover region constant. Both the particle and the spillover region were analyzed.

We analyzed the data using linear mixed effects models in R with the lme4 package (Bates et al., 2015). The three different reading time measures were log transformed to correct for a right skew in the data. The data were analyzed in the same way as in the sentence-matching experiments (see section “Analysis”).

### Results

**FIGURE 3:**
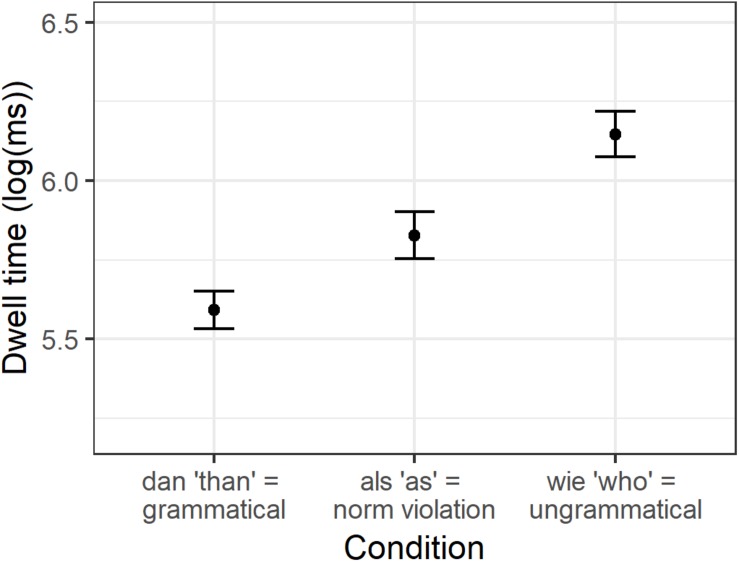
Condition means for logarithmically transformed dwell time on the particle.

The condition means are shown in Table 3. No significant effects were found for first run dwell time. For regression path duration, we found a significant increase in reading time for *wie* compared to *als* (beta = 0.17, SE = 0.06, *t* = 2.940, *p* = 0.006), but not for *als* compared to *dan* (beta = −0.04, SE = 0.05, *t* = −0.875, *p* = 0.38). For dwell time, however, we found that both *dan* (beta = −0.22, SE = 0.05, *t* = −4.311, *p* < 0.001) and *wie* (beta = 0.34, SE = 0.05, *t* = 7.359, *p* < 0.001) differed significantly from *als* (see Figure 3). For the spillover region, we found that first run dwell time was significantly higher after *als* compared to *dan* (beta = −0.09, SE = 0.03, *t* = −2.504, *p* = 0.02), and after *wie* compared to *als* (beta = 0.09, SE = 0.03, *t* = 2.715, *p* = 0.008). The results for regression path duration were again rather similar, with an increase after *als* compared to *dan* (beta = −0.27, SE = 0.05, *t* = −5.34, *p* < 0.001) and an increase after *wie* compared to *als* (beta = 0.33, SE = 0.07, *t* = 4.761, *p* < 0.001). And finally, we found the same results again for dwell time on the spillover region: *als* led to an increase compared to *dan* (beta = −0.28, SE = 0.04, *t* = −6.543, *p* < 0.001) and *wie* led to an increase compared to *als* (beta = 0.36, SE = 0.04, *t* = 8.074, *p* < 0.001).

**TABLE 3:** Condition means and SDs in milliseconds on particle and spillover region for first run dwell time, regression path duration, and dwell time.

	**Reading time measure**					
	**First run dwell time**		**Regression path duration**		**Dwell time**	
	***M***	***SD***	***M***	***SD***	***M***	***SD***
**Particle**						
*dan*	244	127	370	310	308	182
*als*	259	143	392	329	427	343
*wie*	266	135	474	405	606	501
**Spillover**						
*dan*	235	126	359	336	312	196
*als*	260	141	509	531	439	328
*wie*	296	196	748	725	675	546

To sum up, the earliest effect was found on the comparative particle for regression path duration: ungrammatical *wie* took significantly longer than the norm violation *als*, but we found no difference for *als* compared to grammatical *dan.* For dwell time on the particle, we found that both *dan* and *wie* differed significantly from *als.* This finding persisted for the spillover region for all three reading times: *als* leads to an increase in reading time compared to *dan*, but *wie* slows reading down even more.

#### Gauging Individual Differences

**FIGURE 4:**
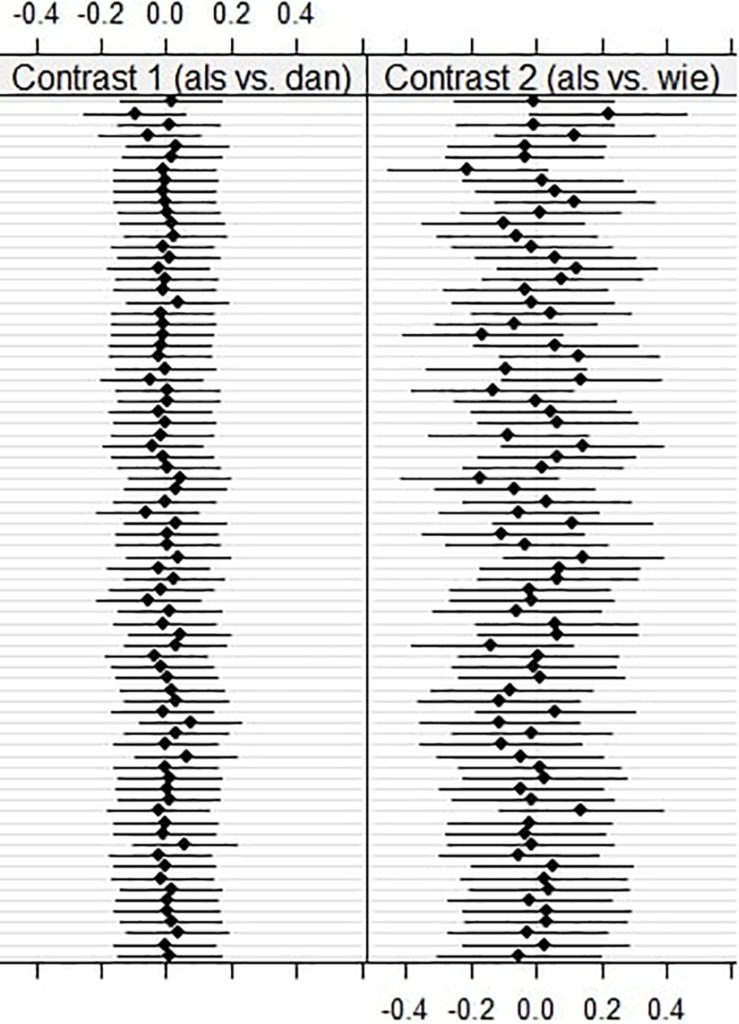
Plots for conditional modes and 95% prediction intervals of all 78 participants for random slopes of both tested contrasts for first run dwell time on the spillover region (see Kliegl et al., 2011).

As can be seen in Table 4, we found that random slopes per participant for the contrast comparing *als* and *wie* significantly improved the model for regression path duration on the particle itself, as well as for first run dwell time and regression path duration in the spillover region. Thus, for earlier reading times we found significant individual variation regarding the effect of *als* compared to *wie.* Note that we did not find significant individual variation for first run dwell time on the particle, but this is not surprising, given that we found no significant effects whatsoever for this model. This suggests that participants showed larger individual variation regarding the comparison between *als* and *wie*, while no such individual differences were found for the contrast comparing *als* and *dan*. Figure 4 shows this individual variation for first run dwell time on the spillover region.

**TABLE 4:** Results of significance tests for random slope components per participant.

	**Reading time measure**					
	**First run dwell time**		**Regression path duration**		**Dwell time**	
	**Chi squared (*df* = 1)**	***p*-value**	**Chi squared (*df* = 1)**	***p*-value**	**Chi squared (*df* = 1)**	***p*-value**
**Particle**						
*Contrast 1 (als vs. dan)*	0	1	0	1	0	1
*Contrast 2 (als vs. wie)*	1.883	0.170	4.911	0.027*	0	1
**Spillover**						
*Contrast 1 (als vs. dan)*	0.426	0.514	0.132	0.717	1	0
*Contrast 2 (als vs. wie)*	4.983	0.026*	5.304	0.021*	1.498	0.221

### Discussion

By employing the eye-tracking method, we hoped to gain more insight into the immediate processing of grammatical norm violations compared to a grammatical and ungrammatical alternative, as well as potential changes over time. The earliest significant effect which surfaced suggests that ungrammatical *wie* “who” leads to an increase in processing effort compared to the grammatical norm violation *als* “as.” Interestingly, the difference between grammatical *dan* “than” and the grammatical norm violation *als* “as” was not yet significant in this model. Later on in processing, however, we consistently found that the grammatical norm violation leads to higher reading times than its grammatical counterpart, while the ungrammatical variant leads to higher reading times than the grammatical norm violation. The grammatical norm violation seems to fall in between the grammatical and the ungrammatical alternative. Furthermore, we found that our participants showed large individual variation regarding the comparison between the grammatical norm violation and the ungrammatical alternative, but not for the comparison between the grammatical option and the grammatical norm violation. In other words, while on a group level, ungrammatical *wie* “who” led to higher reading times than the grammatical norm violation *als* “as”, the size of this effect differed vastly between participants. For some participants, *als* “as” was in fact just as bad as *wie* “who”. The effect that the grammatical norm violation led to increased processing compared to the grammatical alternative was consistent across participants.

The earliest effect was found on the particle itself, and in this initial stage we did not find any difference between the grammatical particle *dan* “than” and the prescriptively incorrect particle *als* “as”, whereas the ungrammatical particle *wie* “who” gave rise to an increase in processing cost. One might wonder whether in this initial stage, the sentence is simply not yet ungrammatical, since adding a comparative phrase is not obligatory, and *als* “as” could also be used to introduce a conditional adjunct clause, as in (7).


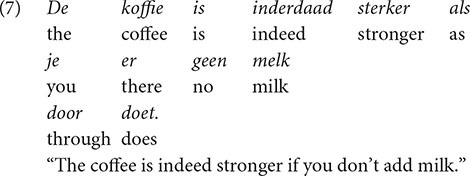


Notoriously, however, people do not read words in an isolated fashion, but are able to preview the upcoming characters and word(s) (e.g., McConkie and Rayner, 1975). Thus, our participants are likely to already have parsed the grammatical norm violation *sterker als gisteren* “lit. stronger as yesterday” as it was intended when we measure their reading time on *als* “as”. This could be seen as weak evidence that participants do indeed process the grammatical norm violation as grammatical and not as ungrammatical in the initial stage of processing. Later on, their processing of the grammatical norm violation falls in between the processing of grammatical and ungrammatical alternatives. This is in accordance with Hubers et al.’s (2016) findings, who assume that processing grammatical norm violations is partly like processing grammatical sentences because both are perfectly interpretable, and partly like processing ungrammatical sentences because they violate a grammatical rule, irrespective of whether the source of the rule is grammar-internal or grammar-external (prescriptive).

## General Discussion

This study investigated the processing of a salient grammatical norm violation in Dutch and German, whether native speakers process such grammatical norm violations as underlyingly grammatical, as ungrammatical, or as somewhere in between. In a series of acceptability judgment tests, Vogel (2019) found that students of German judged grammatical norm violations as equally marked as linguistically marked expressions, that is, in between grammatical and ungrammatical sentences. However, participants showed less uniformity in their judgments of grammatical norm violations than in those of linguistically marked expressions. Hubers et al. (2016) conducted an fMRI study and concluded that grammatical norm violations were processed differently from both grammatical and ungrammatical sentences. The participants in their study were between 30 and 50 years old, and especially selected because they strongly disapproved of grammatical norm violations. It could be that younger speakers who may not hold such a very strong view on grammatical norm violations or who even use these constructions themselves, process them as underlyingly grammatical. This could also be predicted on the basis of Duffield et al. (2002, 2007) who found that certain constructions in French, which were judged ungrammatical in grammaticality judgment tasks, were processed like grammatical sentences in a sentence-matching experiment, which led them to conclude that they were underlyingly grammatical. The authors concluded from this that a sentence-matching task may be a more reliable tool than a traditional grammaticality judgment task in revealing the grammaticality of a construction.

Two versions of the same sentence-matching experiment, a Dutch and a German one, were carried out with university students in the Netherlands and Germany. We hypothesized that the participants would process the grammatical norm violation under consideration as in between grammatical and ungrammatical, as expected on the basis of Hubers et al.’s (2016) fMRI study, and in line with Vogel’s (2019) acceptability judgment tests, but not in accordance with Duffield et al. (2002, 2007) findings that certain constructions that are judged ungrammatical are processed like grammatical ones. Strikingly, however, the results of the sentence-matching experiments showed no difference at all between the processing of grammatical norm violations and ungrammatical sentences, whereas there was a clear difference between the grammatical norm violations and the grammatical sentences. Hence, for the Dutch and German university students in our experiment, processing a grammatical norm violation is just as problematic as processing an ungrammatical sentence. There was no indication whatsoever that grammatical norm violations could be considered underlyingly grammatical.

Various linguistic case studies have shown the influence of prescriptive grammar rules on language use and language change unambiguously (e.g., Hubers and de Hoop, 2013; Hinrichs et al., 2015). Yet, linguists generally consider grammatical norm violations to be grammatical, simply because they frequently occur in the language under consideration, which means they can be generated and understood by the grammatical system. By contrast, the majority of the speakers of a language, in particular educated speakers such as the university students used in our experiments, may be convinced that grammatical norm violations are ungrammatical, as this is what they have learned in school or at home. Vogel (2019: 48) calls this a “paradox”: a grammatical norm violation is generated by the principles of the grammar, otherwise it would not occur at all in the language, but because it violates socially induced grammatical norms, speakers believe that the construction cannot (or should not) be part of that grammar. The linguistic awareness of prescriptive grammar rules may account for the fact that the participants processed grammatical norm violations in the same way as ungrammatical sentences in the sentence-matching experiment. Because a sentence-matching task is a purely linguistic task, participants are very much focused on the grammatical form of the sentence. Also, the decision that has to be made in a sentence-matching experiment, namely, whether the second sentence is identical to the first or not, requires careful consideration of the entire sentence. In the final stage of processing, when they have to make the decision, participants will generally be aware of the presence of a grammatical norm violation, which they have read even twice in a row (because the two experimental sentences were always identical in the task). Supposedly, this explains their delay in reaction time. Note that this type of processing does not reflect what is going on in everyday speech, where grammatical norm violations may often remain unnoticed, because they occur rather frequently, and because they are perfectly interpretable.

In order to find out whether grammatical norm violations are being processed as grammatical or ungrammatical in a somewhat more natural type of setting, we conducted an eye-tracking experiment. In this experiment, sentences were only presented once, and there was no additional linguistic task. Besides, unlike in the sentence-matching experiment, the processing was measured incrementally, that is, right from the beginning of the reading process. Here we found an immediate difference between the processing of ungrammatical sentences and grammatical norm violations. In the very first stage of processing, we did not find a difference between the processing of grammatical norm violations and that of grammatical sentences, while ungrammatical sentences did already lead to an increase in processing cost. After this initial stage of processing, the grammatical norm violation in the eye-tracking study consequently behaved in between grammatical and ungrammatical sentences, confirming the findings of Hubers et al. (2016) as well as Vogel (2019). Also, we found more variation among the participants regarding the difference between the grammatical norm violation and the ungrammatical alternative. No significant individual differences were found for the comparison between the grammatical norm violation and the grammatical alternative. The first encounter with the grammatical norm violation did not lead to a significant increase in processing cost, unlike ungrammaticality. However, after this initial stage processing difficulties do occur, although overall less severe than in the case of the ungrammatical alternative. For some participants, however, the grammatical norm violation is just as bad as the ungrammatical sentence, as reflected in individual differences.

## Conclusion

The aim of this paper was to shed light on the processing of grammatical norm violations or grammatical taboos (Hubers et al., 2016; Vogel, 2019). We focused on one grammatical norm violation in particular, namely, the use of an equative particle in a comparative construction, which occurs frequently in Dutch as well as German, and which is well-known for being a violation of prescriptive grammar rules. We investigated whether this grammatical norm violation gets processed as grammatical or ungrammatical or as something in between.

The results of two sentence-matching experiments, one in Dutch and one in German, indicated that the grammatical norm violation was processed as ungrammatical. However, we hypothesized that this might be explained by the fact that in a sentence-matching task processing is only measured after the full sentence has been taken into account, at which point participants are probably fully aware of the grammatical norm violation they have encountered.

Evidence for this hypothesis was obtained from an eye-tracking experiment, in which the difference between the grammatical norm violation and the ungrammatical alternative immediately surfaced, while we did not find a difference in processing between the grammatical norm violation and its grammatical counterpart early on. Later on in processing, the grammatical norm violation consistently fell in between the grammatical and ungrammatical variants. Moreover, the difference between the grammatical norm violation and the ungrammatical alternative showed a large amount of individual variation, suggesting that for some language users the grammatical norm violation was just as bad as the ungrammatical alternative.

## Data Availability Statement

The datasets generated for this study are available on request to the corresponding author.

## Ethics Statement

The study was reviewed and approved by the Ethics Assessment Committee (EAC) of the Faculty of Arts and the Faculty of Philosophy, Theology and Religious Studies of Radboud University Nijmegen (numbers 6889 and 4592), in line with the Declaration of Helsinki.

## Author Contributions

All authors contributed to the conception and design of the study, manuscript revision, and read and approved the submitted version. FH, TR, and HV were involved in the data collection. FH, TR, and HH wrote the manuscript. FH and TR performed the statistical analyses.

## Conflict of Interest

The authors declare that the research was conducted in the absence of any commercial or financial relationships that could be construed as a potential conflict of interest.

## Footnotes

1 The news item on this can be found online at: http://www.rtlnieuws.nl/editienl/laatste-videos-editienl/wnl-corrigeert-taalfoutje-van-miltenburg.
