# Supplementary material for: Processing Prescriptively Incorrect Comparative Particles: Evidence From Sentence-Matching and Eye-Tracking
Source: Front Psychol. 2020 Feb 14;11:186. doi: 10.3389/fpsyg.2020.00186 (PMC7034421; doi:10.3389/fpsyg.2020.00186)
Supplement: Supplementary file 1 [file Table_1.DOCX]

Supplementary Material

Processing Prescriptively Incorrect Comparative Particles: Evidence from Sentence-Matching and Eye-Tracking

**Ferdy Hubers, Theresa Redl*, Hugo de Vos, Lukas Reinarz, Helen de Hoop**

*** Correspondence:** Theresa Redl: Theresa.Redl@mpi.nl

| **Supplementary Table S1.** Condition means and SDs in milliseconds in Experiment 1 | | | |
| --- | --- | --- | --- |
|  | ***M*** | ***SD*** |  |
|  |  |  |  |
| Grammatical *dan* | 1483 | 412 |  |
| Norm violation *als* | 1619 | 368 |  |
| Ungrammatical *wie* | 1627 | 406 |  |

| **Supplementary Table S2.** Condition means and SDs in milliseconds in Experiment 2 | | | |
| --- | --- | --- | --- |
|  | ***M*** | ***SD*** |  |
|  |  |  |  |
| Grammatical *dan* | 1551 | 374 |  |
| Norm violation *als* | 1623 | 409 |  |
| Ungrammatical *wie* | 1646 | 412 |  |
